# Symptom Burden and Treatment Satisfaction Among Patients with Cancer in a Day Care Unit

**DOI:** 10.3390/medicina62040656

**Published:** 2026-03-30

**Authors:** Anastasia Gavala, Stella Ploukou, Ioanna Tsatsou, Maria Saridi, Pavlos Sarafis, Ourania Govina, Theocharis I. Konstantinidis

**Affiliations:** 1 Medical Oncology Department, Venizelio–Pananio General Hospital, 71409 Heraklion, Greece; angavala@gmail.com; 2 Theagenio Cancer Hospital of Thessaloniki, 54639 Thessaloniki, Greece; sploukou@auth.gr; 3 Department of Nursing, University of West Attica, 12243 Egaleo, Greece; ugovina@uniwa.gr; 4 Department of Nursing, University of Thessaly, 41500 Larissa, Greece; msaridi@uth.gr (M.S.); psarafis@uth.gr (P.S.); 5 Department of Nursing, Hellenic Mediterranean University, 71410 Heraklion, Greece; harriskon@hmu.gr

**Keywords:** BFI, chemotherapy, CTSQ, fatigue, MDASI, patients, satisfaction, symptoms

## Abstract

*Background/Objectives:* Patients with cancer undergoing chemotherapy experience multiple symptoms. Effective identification, assessment, and management of these symptoms improve treatment effectiveness and satisfaction, as well as their quality of life. The present study aimed to record the symptoms of cancer patients and assess their satisfaction with chemotherapy in a Day Care Unit. *Materials and Methods:* This cross-sectional study involved 95 cancer patients under chemotherapy. Data were collected using the Greek M.D. Anderson Symptom Inventory, the Brief Fatigue Inventory, the Cancer Treatment Satisfaction Questionnaire, and a sociodemographic questionnaire. Statistical analysis was performed using software SPSS 26.0. *Results:* Participants had a mean age of 63.8 ± 10.2 years, were mainly married (75.8%) and male (50.5%), and had a mean time since diagnosis of 1.9 years. Fatigue was the most common moderate symptom (81.1%, M = 4.26), whereas vomiting (18.9%, M = 1.03) and dyspnea (22.1%, M = 1.03) were infrequent and mild. Fatigue was significantly associated with Satisfaction with Therapy (SWT) and symptom severity. Overall satisfaction was moderate to high, with the Feelings About Side Effects (FSE) subscale scoring lowest (M = 54.0, SD = 24.7); females had lower odds of very high SWT than males (OR = 0.10, *p* = 0.003). Higher SWT was associated with being married (rho = −0.260, *p* < 0.05) and having higher education (rho = 0.276, *p* < 0.05). Higher levels of education were also associated with lower fatigue (rho = −0.233, *p* < 0.05), while positive FSE and higher Expectations of Therapy were linked to fewer severe symptoms and reduced fatigue, respectively. *Conclusions:* Cancer patients receiving chemotherapy reported low symptom severity and moderate to high SWT, although fatigue remained the most prominent and impactful symptom. These findings emphasize the importance of systematic symptom monitoring, particularly fatigue management, to enhance patient satisfaction and optimize the overall treatment experience.

## 1. Introduction

Cancer is a leading cause of morbidity and mortality worldwide, with its incidence projected to rise by over 76.6% from 2022 to 2050, reaching over 35.3 million new cases annually [1]. Environmental exposures, unhealthy lifestyle choices (junk food and tobacco use), and an aging population are the main causes of the current trend [1]. Thankfully, survival rates are improving due to advances in screening and treatment, such as chemotherapy. Still, healthcare systems must balance treatment effectiveness with a focus on chronic symptoms that affect patients’ quality of life [2]. Chemotherapy poses a particular burden on patients on both physical and psychological levels. Symptoms such as fatigue, nausea, discomfort, and cognitive deficits are common and may persist long after treatment has ended, affecting satisfaction with treatment [3].

The aggressiveness of chemotherapy regimens can vary significantly depending on the specific cancer type and stage of the disease [4]. While early-stage malignancies may require adjuvant protocols aimed at eradication, more advanced stages often involve intensive or long-term systemic therapies to manage disease progression [5]. These aggressive treatments are often linked to a cumulative symptom burden [6], with higher toxicity levels leading to more severe physical and psychological distress [7]. Consequently, patients’ experiences and their ability to tolerate treatment are deeply influenced by the clinical complexity of their diagnosis and the resulting intensity of the therapeutic intervention.

Fatigue is a persistent symptom that tends to be more severe and last longer in cancer patients than in non-cancer patients [8]. Research indicates that these symptoms interfere with everyday functioning, emotional well-being, and socialization, emphasizing the importance of the patient experience during chemotherapy [2,9].

The assessment of treatment satisfaction is increasingly recognized as a vital patient-reported outcome (PRO) that reflects the quality of oncology services from the patient’s perspective [10]. Unlike clinical indicators alone, satisfaction metrics provide a more comprehensive view of the care experience, including the effectiveness of communication, management of toxicities, and patient trust in the therapeutic regimen [11]. Understanding these nuances is particularly critical in Day Care Units (DCUs), where the outpatient nature of chemotherapy means patients must manage significant side effects at home [12]. By identifying the specific factors that drive or diminish satisfaction, healthcare providers can implement evidence-based changes to care delivery that improve patient well-being and enhance long-term outcomes.

Understanding patients’ personal experiences, particularly their satisfaction with therapy, is essential for improving clinical outcomes [2,13]. High levels of patient satisfaction are associated with better treatment outcomes, reduced psychological distress, improved self-management, and a higher quality of life [14,15,16]. Recording and assessing patient reports on symptoms and satisfaction with chemotherapy as part of routine oncology care can lead to tailored interventions, strengthen clinician–patient relationships, and establish holistic care pathways that align with survival and well-being goals [17,18].

This study aimed to evaluate symptom burden, fatigue levels and treatment satisfaction among cancer patients undergoing chemotherapy in a DCU, examining their associations with patients’ demographic and clinical characteristics.

## 2. Materials and Methods

### 2.1. Study Design and Setting

This exploratory, observational cross-sectional study was conducted at a DCU of a regional general hospital in the 7th Health District of Crete, from April to September 2021.

### 2.2. Participants and Sampling

**Figure 1:**
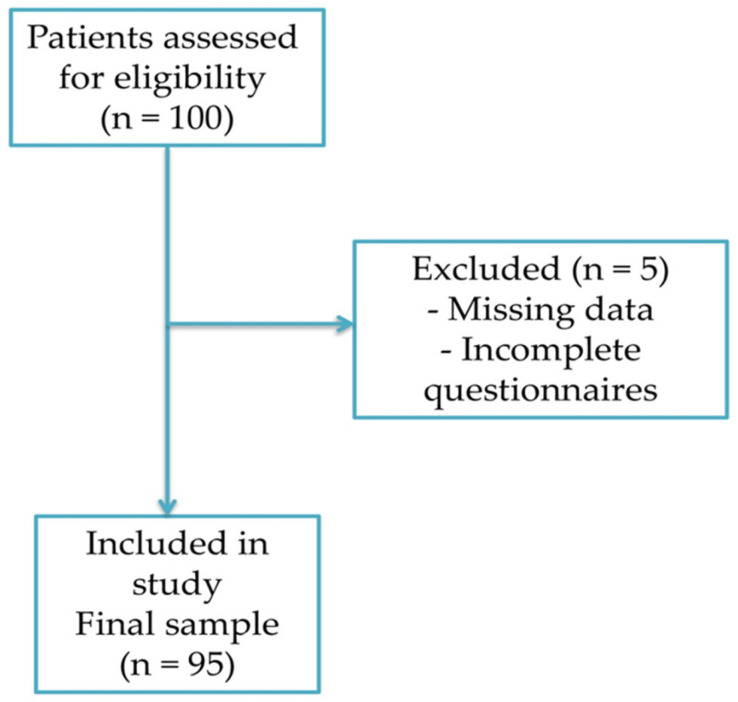
Participant Selection Flow Diagram.

Initially, 100 patients were approached for participation using convenience sampling from among those hospitalized for active treatment in the DCU. Recruitment was limited by practical and clinical factors, such as patients’ disease burden, treatment schedules, and their willingness or ability to participate. Five patients were excluded due to missing health history data or incomplete responses on the assessment scales. The final study sample consisted of 95 cancer patients undergoing chemotherapy at the DCU (Figure 1). Inclusion criteria comprised adults (aged ≥18 years) diagnosed with cancer and undergoing chemotherapy, regardless of cancer type, disease stage, or type of chemotherapy treatment. As the sample size was determined by the total number of eligible patients who consented to participate during the study period in this specific clinical setting, no a priori sample size or statistical power calculation was performed. Therefore, this study should be considered exploratory and its findings should be interpreted with appropriate caution.

### 2.3. Data Collection

The study used four questionnaires to collect data on fatigue, satisfaction, and cancer-related symptoms in cancer patients. Patients completed a 12-question survey covering demographic and clinical data, the Brief Fatigue Inventory (BFI), Cancer Therapy Satisfaction Questionnaire (CTSQ) and the M.D. Anderson Symptom Inventory.

The Greek validated version of the BFI questionnaire was used [19] to measure fatigue severity. The validity and reliability of the original scale have been documented. The questionnaire uses an 11-point scale (0 = “no fatigue” to 10 = “the worst fatigue you have ever felt”). Three items ask patients to rate the severity of their fatigue at “worst,” “usual,” and “now” over the past 24 h. A score of ≥7 indicates “severe fatigue” and 0–6 indicates “non-severe.” Six additional items describe how fatigue interferes with different aspects of the patient’s life over the past 24 h. These items include general activity, mood, ability to walk, normal work, relationships with other people, and enjoyment of life. This is measured with 0 = “does not interfere” and 10 = “interferes completely.” The total score for the BFI is calculated as the average of these nine items.

The CTSQ assesses patient satisfaction with treatment [20]. The Greek validated version was used [16]. It consists of three dimensions that assess expectations from treatment (5 questions), feelings about side effects (4 questions), and cancer patients’ satisfaction with treatment (7 questions), regardless of the type, stage of cancer, and type of chemotherapy they are undergoing. Each question is scored from one to five, with the lowest score representing the worst response. Four questions are reversed, and the score for each dimension is calculated using the formula: (mean dimension score −1) × 25. The result of each dimension’s score ranges from 0 to 100, with the highest score representing more positive results.

The Greek M.D. Anderson Symptom Inventory (G-MDASI) is a self-administered questionnaire that assesses the severity and impact of cancer-related symptoms and the extent to which symptoms affect a patient’s feelings and functioning over the past 24 h [21]. It is a self-administered questionnaire that provides a brief assessment of the severity and impact of cancer-related symptoms. The first part includes 15 symptoms (pain, fatigue, nausea, vomiting, sleep disturbances, anxiety, dry mouth, sadness, etc., in the last 24 h), each of which is rated on an 11-point Likert severity scale from 0 to 10 (where 0 = “the symptom does not affect me” and 10 = “the symptom has affected me severely”). The second part assesses how these symptoms affect how the patient felt and how their functioning was affected in the last 24 h. It includes 6 questions (general activity, mood, etc.). No specific cut-off points were applied for the G-MDASI scales in this study. All items were analyzed as continuous variables according to the original scoring guidelines of the instrument, and no modifications to the validated Greek version were made.

The research team consisted of two trained oncology nurses who also served as clinical researchers. Both of them underwent a standardized training session prior to data collection, which focused on the consistent application of the inclusion criteria and the appropriate administration of the study’s questionnaires. Researchers were also trained to provide uniform verbal briefings on the study’s objectives and the informed consent process to ensure participant comprehension and ethical compliance.

### 2.4. Data Analysis

Data analysis was performed using SPSS Statistics 26.0 (IBM Corp., Released 2019, IBM SPSS Statistics for Windows, v.26.0, Armonk, NY, USA: IBM Corp.), which was selected for its widespread use in medical research and its suitability for descriptive, nonparametric, and multiple logistic regression analyses. Because the data were not normally distributed, nonparametric statistical methods were primarily used; logistic regression was additionally employed where appropriate to model categorical outcomes. Frequency distributions of descriptive characteristics, disease status, and comorbidities among participants were calculated with appropriate consideration of 95% confidence intervals for comparability reasons. The shapes of the distributions of the Fatigue (BFI-Gr), Satisfaction (CTSQ), and Symptom Measurement (G-MDASI) scales’ scores were checked using the Blom method (QQplot), while reliability coefficients were calculated using the Cronbach method, where applicable. Due to asymmetry across most scales/subscales, the non-parametric Spearman correlation method was used to assess correlations among them and with patients’ basic characteristics. The non-parametric Kruskal–Wallis test was also used to compare their scores. Multiple logistic regression was used to assess the association (odds ratio, OR) between high and moderate/low treatment satisfaction and fatigue, symptoms, and participants’ descriptive characteristics. The significance level was set at 0.05.

### 2.5. Ethics

The study was conducted in accordance with the Helsinki Declaration for medical research involving human subjects and approved by the General Hospital of Iraklion (protocol number 5-22/4/2021) and the Department of Nursing, Hellenic Mediterranean University Ethics Committee (protocol number 491-8/3/2021).

All participants received a verbal briefing on the study’s objectives, procedures, and methods before signing informed consent. Patients were clearly informed that they would not receive compensation for their participation and could withdraw at any time. Participants’ personal information was anonymized to ensure their safety during the study.

## 3. Results

### 3.1. Participant Characteristics

The participants’ mean age was 63.8 ± 10.2 years; the majority were male (50.5%) and married (75.8%) and lived with family members in the wider Heraklion area (80.0%). Of the participants, 16.8% had attained university-level education, while 31.6% were actively employed. The average time since cancer diagnosis was 1.9 years. Cancer-related surgery had been performed on 62.1% of participants, and radiotherapy on 17.9%. Additionally, seven participants (7.4%) had multimorbidity, with cardiovascular disease being the most frequent comorbidity, affecting 16.8% of the group (Table 1).

### 3.2. Fatigue

**Figure 2:**
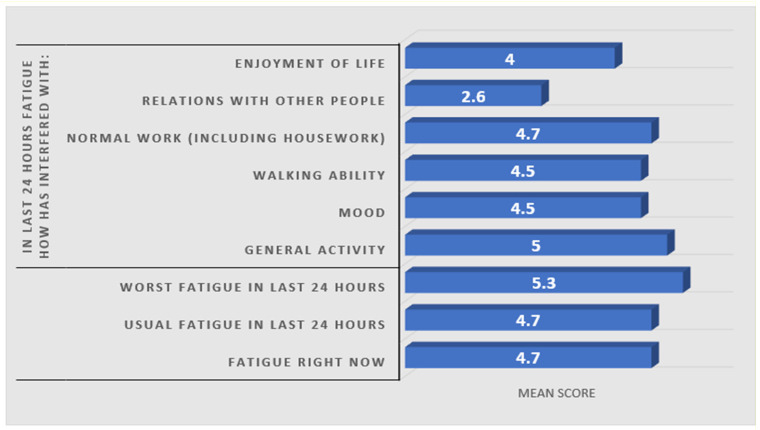
BFI-Gr scale mean scores of the 95 participants. Mean scores of 9 questions on a scale of 0–10, where 0: no fatigue-does not interfere and 10: as bad as you can imagine-completely interferes.

Most patients (85.3%) reported experiencing fatigue during the previous week (*p* < 0.05). The mean total score on the BFI-Gr was 40.0 (SD = 22.4), indicating moderate-to-low fatigue levels. Severe fatigue, defined as a score of >63 by Mystakidou et al. 2008 [19], was observed in 16 patients (Table 2). The highest mean score was reported among the BFI-Gr items for worst fatigue in the last 24 h (M = 5.3). In contrast, the lowest mean score was reported for the impact of fatigue on relationships with others (M = 2.6), with a maximum score of 10 indicating the worst fatigue (Figure 2).

### 3.3. Therapy Satisfaction

Using the CTSQ, patients reported moderate to high levels of satisfaction. The highest mean score was observed on the Satisfaction with Therapy (SWT) subscale (M = 82.2, SD = 13.5), and the lowest on the Feelings about Side Effects (FSE) subscale (M = 54.0, SD = 24.7). Very high levels of SWT (>75) were reported by 75.8% of patients, very high Expectations of Therapy (ET) (>75) by 70.5%, and very positive FSE by 24.2% (Table 2).

### 3.4. Symptom Burden

The overall symptom intensity, as measured by the G-MDASI, was low (M = 2.6, SD = 2.0). Fatigue emerged as the most frequent and intense core symptom (81.1% of patients, M = 4.26). Conversely, vomiting (18.9%) and shortness of breath (22.1%) were the least reported symptoms. Among the interference symptoms, walking had the highest mean impact (73.7%, M = 3.88), while social relationships had the lowest (43.2%, M = 2.36) (Table 2 and Table 3).

### 3.5. Correlations Between Variables

Table 4 presents the univariate correlations of the 95 patients across the three measurement scales. Significant correlations between BFI-Gr, G-MDASI and CTSQ were observed in almost all the controls. Overall, an increase in the intensity of the G-MDASI was associated with an increase in Fatigue (rho = 0.788, *p* < 0.05) and with a decrease in ET (rho = −0.338, *p* < 0.05), FSE (rho = −0.573, *p* < 0.05) and SWT (rho= −0.574, *p* < 0.05). Similarly, an increase in patients’ Fatigue appears to be associated with a decrease in ET (rho= −0.394, *p* < 0.05), FSE (rho= −0.464, *p* < 0.05) and SWT (rho= −0.481, *p* < 0.05).

### 3.6. Correlations of BFI-Gr, G-MDASI, and CTSQ Scales with Patients’ Demographic and Clinical Characteristics (n = 95)

Significant associations were found for gender, education, marital status, and years since diagnosis. Among other findings, male gender appears to be associated with higher levels of positive FSE (rho = −0.288, *p* < 0.05) or SWT (rho = −0.373, *p* < 0.05) and lower symptom intensity measured with G-MDASI (rho = 0.261, *p* < 0.05). Married people were also associated with higher levels of SWT (rho = −0.260, *p* < 0.05). Higher levels of education were associated with lower Fatigue (rho = −0.233, *p* < 0.05), higher SWT (rho = 0.276, *p* < 0.05) and lower overall symptom severity as measured using the G-MDASI (rho = −0.278, *p* < 0.05). More years since diagnosis were also associated with higher Fatigue (rho = 0.236, *p* < 0.05) or lower ET (rho = −0.290, *p* < 0.05) and lower positive FSE (rho = −0.229, *p* < 0.05) (Table 5).

### 3.7. Multiple Analysis

Table 6 presents the results of the multiple logistic regression analysis, expressed as adjusted odds ratios (ORs), comparing high versus moderate/low CTSQ scores with respect to BFI-Gr, G-MDASI, and selected demographic and clinical characteristics of the 95 patients. The variables included in the models (sex, marital status, education level, years from diagnosis, G-MDASI, and BFI-Gr) were selected a priori based on their clinical relevance and potential role as confounders. Since CTSQ comprises three components (ET, FSE, and SWT), the evaluation was conducted separately for each component using characteristics found to be interrelated. Specifically, for every unit increase in the BFI-Gr score, the odds of having very high ET decrease (OR = 0.96, *p* = 0.050). Similarly, for every unit increase in the G-MDASI score, the odds of very positive FSE decrease significantly (OR = 0.45, *p* = 0.018), while women have significantly lower odds of very high SWT compared to men (OR = 0.10, *p* = 0.003).

These findings suggest that lower fatigue levels are associated with higher expectations of therapy, and lower symptom burden is associated with more positive feelings about side effects. In addition, male patients appear to report higher levels of satisfaction with therapy.

## 4. Discussion

In the current study, satisfaction with treatment and the severity and impact of cancer-related symptoms were assessed among cancer patients undergoing chemotherapy at a DCU. A negative correlation was found between fatigue and the CTSQ scale, and men reported higher SWT levels than women.

Cancer-related fatigue is a complex phenomenon that changes over time. It is characterized by psychological distress, impaired cognitive function, and an imbalance in energy regulation [22,23]. In line with previous studies, fatigue was identified as the most prevalent symptom, with moderate-to-low levels significantly impacting patients’ daily functioning and overall quality of life [24]. The findings also reveal that a substantial percentage of patients reported severe fatigue, which was strongly correlated with the intensity of other symptoms and negatively impacted patients’ expectations, emotional responses to treatment, and overall satisfaction. The persistence of fatigue even during treatment highlights the importance of its early detection and comprehensive management to enhance patient-centered care and improve the therapeutic experience [25].

The prevalence of cancer-related fatigue varies widely, with estimates ranging from 60% to 90% depending on the diagnostic criteria applied. It often coexists with other distressing symptoms, such as depression, weakness, pain, loss of appetite, insomnia, anxiety, nausea, and breathing difficulties, which may contribute to its intensity [19,23]. Notably, it disrupts everyday functioning and profoundly affects many aspects of patients’ quality of life, including their ability to adhere to treatment plans [22,26]. Given its significant impact on treatment adherence and daily life, oncology teams are encouraged to incorporate standardized, longitudinal fatigue screening into routine clinical practice, enabling early, personalized supportive interventions.

The current group had a lower mean BFI score than that reported by Muthanna et al. (2021) among 172 breast cancer patients receiving chemotherapy (46.5 ± 11.4 vs. 40.0 ± 22.4) [27]. Although not investigated in the present study, stage of malignancy and ethnicity were correlated with the presence of fatigue, as were delays or reductions in chemotherapy dosage. Similarly, a 2015 study in Boston involving 180 patients with various types of cancer receiving chemotherapy found that 25.0% of participants experienced moderate to severe fatigue at baseline [28]. This subgroup reported significantly higher levels of anxiety and depression, as well as poorer quality of life. Other studies have documented varying rates of severe fatigue: 17.4% in a sample of 121 patients in Indonesia [29], 17% in a sample of 206 patients in the Philippines [30], and a notably lower prevalence of 2.1% in a sample of 48 chemotherapy recipients in Italy [31]. These findings, combined with the comparatively modest rates of severe fatigue identified in the present study, are reassuring. They emphasize the importance of systematic assessment and monitoring, which can help oncology teams diagnose treatment-related fatigue early and implement timely interventions to lessen its impact on patients’ quality of life.

Stylianou et al. (2021) conducted a study using the CTSQ that examined SWT in relation to demographic characteristics and symptom severity among 100 chemotherapy patients over six months [16]. Their findings revealed lower mean CTSQ scores than those observed in the present study: ET (60.6 vs. 78.8), FSE (44.6 vs. 54.0), and SWT (75.9 vs. 82.2). Fatigue and anxiety were reported as the most severe symptoms, and all three CTSQ components were significantly associated with symptom variables, including pain, fatigue, anxiety, and nausea. Differences in CTSQ scores have been observed in other studies across various populations. For instance, a Dutch study of 55 lung cancer patients reported lower ET (55.6), FSE (52.2), and SWT (79.7) scores [32]. In contrast, a study of 361 cancer patients of various types and disease stages in the United States showed comparable or higher scores (76.8, 91.9, and 82.8, respectively) [33]. Interestingly, male patients in the current study reported significantly higher SWT scores than female patients. Although this gender difference is not well explained in the literature, some evidence suggests that men, particularly those with gender-specific cancers such as prostate cancer, tend to report greater SWT [34].

Furthermore, the subjective experience of chemotherapy may differ between men and women due to gender-related characteristics. The literature suggests that women may experience higher levels of symptoms and psychological distress, which can affect their overall satisfaction with therapy [35,36]. Additionally, women often have to balance treatment with domestic and childcare or caregiving responsibilities. This means that the impact of symptoms such as fatigue on daily life may be more significant [37,38], as reflected in the high interference scores for activities and housework observed in our study. Our findings emphasize the need for oncology teams to adopt gender-sensitive approaches that address the unique psychosocial needs and symptom burdens of female patients, to bridge this satisfaction gap.

Furthermore, although chemotherapy treatment generally meets expectations, symptoms such as fatigue, anxiety, and pain can still affect satisfaction with treatment. Furthermore, factors relating to the healthcare environment and the delivery of care may play a significant part in shaping patients’ overall treatment experiences. This is important because it suggests that patient satisfaction is a multidimensional concept that goes beyond clinical efficacy. Furthermore, aspects of the healthcare environment and care delivery, such as the quality of communication with oncology nurses and the efficiency of the DCU, can either alleviate or exacerbate the perceived burden of these symptoms. Understanding this interplay is essential for healthcare providers. Focusing solely on survival metrics without addressing environmental and symptomatic experiences may result in lower treatment adherence and poorer psychological well-being. Consequently, improving the physical setting and fostering a supportive care delivery model are as important as the pharmacological treatment itself to the patient experience.

The concepts of positive attitude and hope have been extensively studied among cancer patients. The findings can be summarized as follows: patients with a positive attitude who avoid negativity are more likely to adhere to chemotherapy, resulting in better therapeutic outcomes, which is very important [39,40]. In some cases, knowledge of the consequences of stopping treatment motivated them to adhere to it in the hope that it would make them better [41].

Based on the results of the G-MDASI Symptom Inventory Scale, the Additional I symptom group, which encompasses aspects that interfere with patients’ daily lives, had a significantly higher mean intensity score than the other symptom categories (*p* < 0.001). Nevertheless, the overall mean symptom score was low at 2.4 ± 1.9, which is within the scale’s range of 0 to 10. A score of 10 represents the most severe or complete manifestation of symptoms.

Similar studies using the MDASI have reported variable, but generally low, average symptom scores. For instance, two U.S. studies reported means of 2.2 ± 1.5 [42] and 2.2 ± 1.6 [43], respectively, among 697 and 248 patients, while a Greek study involving 90 patients before radiotherapy initiation found a similar mean of 2.4 ± 1.7 [44]. Fatigue and dry mouth consistently emerged in many studies as the symptoms most affecting patients during treatment [42,43,45,46,47,48]. Although oncology patients tend to report relatively low levels of symptom burden across treatment phases, fatigue remains the most prominent and persistent symptom, prompting scientific interest in improving monitoring and care strategies. Better management of fatigue may enhance treatment adherence and minimize disruption to patients’ daily lives and overall well-being [49].

When interpreting the symptom burden experienced by our sample of patients, it is important to acknowledge that the symptoms reported are likely to be the result of a combination of the underlying malignancy and the secondary effects of various treatment modalities. Although the study assessed patients during chemotherapy, 62.1% of participants had previously undergone surgery and 17.9% had received radiotherapy. Consequently, symptoms such as fatigue (81.1%) and numbness/tingling (60.0%) may be exacerbated by chemotherapy, but could also be influenced by postoperative recovery or the effects of long-term radiation. This complex interplay makes it difficult to identify the cause of each symptom, which is a common issue in oncology research involving patients with multimorbidity or prior interventions. However, by comparing these results with those from regions such as the United States or Northern Europe, where symptom scores and satisfaction levels are often similar despite differing healthcare infrastructures, it becomes apparent that the burden of treatment is a universal challenge for oncology patients, regardless of the specific healthcare system.

As our study focuses on patients in a DCU, it is important to acknowledge the positive impact of the early introduction of palliative care for outpatients and in the community, integrated with oncology care, in recent years. According to recent meta-analyses, palliative care involvement with these patients is negatively associated with mortality and positively associated with short-term quality of life. There is no clear association with long-term quality of life, but there is a small, yet statistically significant, reduction in the burden of physical symptoms [50,51]. A key component of the success of palliative care programmes is the multidisciplinary team, with active nurse participation. This is a service missing in Greece, where the implementation of palliative care is underdeveloped and fragmented [52].

Ultimately, the results of this study contribute to the wider global discussion on optimizing oncology care through public health policy [53]. According to the 2025 OECD report [54], there is currently no centralized, official mechanism for systematically collecting patient-reported outcomes (PROs) across the Greek healthcare system. Most data comes from academic or cross-sectional studies (similar to our research), rather than from routine clinical practice. While some oncology departments and private clinics use PROs, their use is neither standardized nor integrated into electronic health records. The report also highlights the fragmented nature of palliative care in Greece, supporting our call for these services to be integrated into DCUs early on to improve symptom management.

While our findings reflect the specific healthcare environment of a Greek DCU, the literature indicates that patient-reported outcomes can vary significantly across continents due to disparities in economic development, government funding, and healthcare infrastructure. Regions with robust social support and high healthcare expenditure often focus on nuanced quality-of-life interventions. Conversely, structural barriers and limited access to supportive care in developing economies can exacerbate symptom burden [55]. Furthermore, cultural attitudes towards cancer and chemotherapy-induced symptoms can significantly influence how patients perceive and report their satisfaction [56]. By identifying the close link between satisfaction and both clinical symptom management and patient expectations, this study highlights the need for health policies that are both economically viable and culturally appropriate to bridge the gap between treatment efficacy and the patient’s lived experience. Such cross-continental comparisons are essential for developing evidence-based strategies that ensure equitable and compassionate cancer care worldwide.

### Limitations and Strengths

The current study had some limitations. First, using a convenience sample, without stratification by cancer type or disease stage, may limit the generalizability of the findings. An a priori sample size calculation was not performed. The study population was small and consisted of patients receiving treatment in a DCU, where recruitment was limited by practical and clinical constraints, including patients’ disease burden, treatment schedules, and variable willingness or ability to participate. As a result, the final sample represents all eligible patients who consented to participate during the study period. Given these real-world recruitment limitations and the lack of reliable preliminary data from similar populations in the specific clinical context, any assumptions regarding effect size or statistical power prior to data collection would have been highly uncertain. Given the number of variables analyzed, the relatively small sample size may also limit the statistical power to detect smaller effect sizes. Therefore, we acknowledge that the absence of an a priori power calculation constitutes a methodological limitation and may introduce potential bias.

Additionally, the cross-sectional design does not permit causal inferences or the evaluation of changes in symptom burden or treatment satisfaction over time. The relatively small sample size, coupled with the lack of stratification by cancer type, disease stage, or treatment regimen, may have obscured potential subgroup differences. Furthermore, data collection during the pandemic may have affected patients’ access to care, psychological status, symptom reporting, and satisfaction. Finally, reliance on self-reported measures may introduce response bias, despite the use of validated instruments.

Despite these limitations, the study used well-validated and reliable questionnaires to comprehensively assess fatigue, symptom burden and treatment satisfaction. Additionally, evaluating clinical, demographic, and psychosocial variables simultaneously provides a multidimensional understanding of the relationship between symptom experience and satisfaction with therapy. The findings offer valuable real-world insights from a DCU setting and contribute meaningful data to the limited Greek literature on patient-reported outcomes in oncology care.

## 5. Practice Implications

This study emphasizes the vital role of healthcare professionals in managing and alleviating the symptoms experienced by cancer patients undergoing chemotherapy. Their responsibilities include providing psychological support and delivering personalized, compassionate, evidence-based interventions that treat patients holistically. For patients, fatigue can result from broader contextual factors such as living conditions, emotional stress, and personal resilience.

Trained oncology nurses play a crucial role in the early identification, prevention and treatment of cancer by implementing standardized nursing care protocols that address all health needs. Simple strategies such as the early integration of palliative care, minimizing hospital stays, tailoring symptom management to cancer type, stage and treatment modality, and adopting a multidisciplinary team approach can significantly improve patient care. These simple yet targeted interventions are easier to implement and are equally effective in maximizing the recovery process.

## 6. Conclusions

This study aimed to document the symptoms experienced by patients with cancer undergoing chemotherapy in a DCU and to assess their satisfaction with the treatment process. The findings suggest that patients generally experienced low overall symptom severity and moderate to high satisfaction levels with therapy. However, despite this generally positive outlook, fatigue was found to be the most common and intense symptom, significantly impacting the overall patient experience and daily functioning.

Perhaps the most important findings concern the assessment that an increase in expectations of treatment is significantly related to a reduction in fatigue and an increase in positive feelings about treatment. There is also a reduction in the intensity or presence of symptoms, and male patients express higher levels of overall satisfaction with treatment. Therefore, managing and reducing symptoms, particularly fatigue, in cancer patients can improve satisfaction with treatment and increase expectations, optimism, and well-being, which are essential for completing treatment and overcoming the disease.

Integrating early palliative care principles that focus on comprehensive symptom management and psychosocial support could further improve these outcomes and enhance overall quality of life. Future research should explore longitudinal outcomes and evaluate targeted fatigue management and supportive care interventions in larger, more diverse cancer patient groups. Given that fatigue and satisfaction with care emerged as crucial concerns, future multicentre studies involving larger sample sizes could consider various factors that influence these outcomes. We hope that the findings of this study will contribute to a deeper understanding of the relationship between symptom burden and treatment satisfaction in patients undergoing chemotherapy. Ultimately, we hope that these results will support the development of more patient-centered, evidence-based interventions that enhance the quality of care and well-being and improve overall treatment outcomes.

## Figures and Tables

**Table 1:** Sociodemographic and clinical characteristics of 95 oncology patients.

Sociodemographic Characteristics	n (%)
Sex	
Male/Female	48 (50.5)/47 (49.5)
Age	
Mean age ± SD (years)	63.8 ± 10.2
Age group 40–60 years	39 (41.1)
Age group 61–85 years	56 (58.9)
Marital Status	
Married	72 (75.8)
Other (single, widowed, divorced)	23 (24.2)
Living Situation	
Living alone	17 (17.9)
Living with others (family, spouse)	83 (82.1)
Level of Education	
Primary	25 (26.3)
Secondary	54 (56.9)
Tertiary	16 (16.8)
Employment Status	
Employed	30 (31.6)
Unemployed, Retired	65 (59.4)
Place of Residence	
Heraklion	76 (80.0)
Other cities	19 (20.0)
Clinical Characteristics	
Time (years) since diagnosis [mean, range]	1.9 (0.1–7.7)
Surgical treatment	59 (62.1)
Radiotherapy	17 (17.9)
Cardiovascular comorbidity	16 (16.8)
Multimorbidity (≥2 comorbid conditions)	7 (7.4)

**Table 2:** Brief Fatigue Inventory, Cancer Therapy Satisfaction Questionnaire and M.D. Anderson Symptom Inventory scores of 95 oncology patients.

Scale	Mean	SD	Cronbach’s α
Fatigue (BFI-Gr) ^1^	40.0	22.4	0.940
Severe fatigue (63+)	n = 16 (16.8%)		
Therapy Satisfaction (CTSQ) ^2^			0.832
Expectations of Therapy (ET)	78.8	19.0	0.838
Very high expectations (75+)	n = 67 (70.5%)		
Feelings about Side Effects (FSE)	54.0	24.7	0.753
Very positive feelings (75+)	n = 23 (24.2%)		
Satisfaction with Therapy (SWT)	82.2	13.5	0.723
Very high satisfaction (75+)	n = 72 (75.8%)		
Symptom Inventory (G-MDASI) ^3^	2.6	2.0	0.936
Basic Symptoms ^4^	2.3	1.9	0.888
Symptom Interference ^5^	3.4	3.0	0.947

^1^ Summarized score of 9 sentences (on a scale of 0–10) where a higher score (→90) indicates higher fatigue. The threshold of 63 refers to the corresponding 7.00 reported by Mystakidou and et al., 2008 [19]. ^2^ Subscale score 0–100, where a higher score indicates higher satisfaction. ^3^ Scale score 0–10, where a higher score indicates higher symptom intensity. ^4^ Response scale from 0: the symptom did not occur to 10: the worst you can imagine. ^5^ Response scale from 0: the symptom did not interfere to 10: the symptom interfered completely. Kruskal–Wallis tests between the 3 satisfaction subscales (*p* < 0.001) & the 4 symptom groups (*p* < 0.001).

**Table 3:** G-MDASI scale scores of the 95 participants.

Symptoms		Mean	SD	%
Symptoms Items ^1^	Pain	2.08	3.12	43.2
	Fatigue	4.26	3.35	81.1
	Nausea	2.37	3.17	46.3
	Disturbed sleep	2.99	3.09	65.3
	Distress/feeling upset	2.47	2.91	58.9
	Shortness of breath	1.03	2.33	22.1
	Difficulty remembering	1.06	2.21	27.4
	Lack of appetite	2.17	3.11	43.2
	Drowsiness	2.57	2.84	61.1
	Dry mouth	2.92	3.24	66.3
	Sadness	2.42	3.38	47.4
	Vomiting	1.03	2.50	18.9
	Numbness/tingling	2.76	3.31	60.0
	Diarrhea	1.66	2.77	36.8
	Constipation	2.43	3.33	49.5
Interference items ^2^	Activity	3.71	3.23	73.7
	Mood	3.58	3.25	73.7
	Working (including housework)	3.57	3.40	69.5
	Relations with other people	2.36	3.25	43.2
	Walking	3.88	3.44	73.7
	Enjoyment of life	3.59	3.40	67.4

^1^ 0 = symptom was not present, to 10 = the symptom was as bad as you can imagine it could be. ^2^ 0 = symptoms have not interfered, to 10 = symptoms interfered completely.

**Table 4:** Selected correlations between key variables.

Variables	rho-Spearman
Fatigue (BFI-Gr) ^1^	
Fatigue (BFI-Gr)—Symptom Intensity (G-MDASI) ^2^	0.788
Fatigue (BFI-Gr)—Expectations of Therapy	−0.394
Fatigue (BFI-Gr)—Feelings about Side Effects	−0.464
Fatigue (BFI-Gr)—Satisfaction with Therapy	−0.481
Therapy Satisfaction (CTSQ) ^3^	
Expectations of Therapy—Feelings about Side Effects	0.239
Expectations of Therapy—Satisfaction with Therapy	0.449
Expectations of Therapy—G-MDASI	−0.338
Feelings about Side Effects—Satisfaction with Therapy	−0.445
Feelings about Side Effects—G-MDASI	−0.573
Satisfaction with Therapy—G-MDASI	−0.574

^1^ A higher score indicates higher fatigue. ^2^ A higher score indicates higher symptom intensity. ^3^ A higher score indicates higher satisfaction. For all above, the correlation *p*-value < 0.05.

**Table 5:** Correlation of the BFI-Gr, CTSQ, and G-MDASI scores of 95 oncology patients regarding their demographic and clinical characteristics.

	Correlations	Sex ^1^	Age ^2^	Marital Status ^3^	Living Status ^4^	Education ^5^	Residence ^6^	Years Since Diagnosis	Multi-Morbidity
Fatigue (BFI-Gr) ^7^	rho	0.136	0.148	0.090	0.076	−0.233 *	0.038	0.236 *	0.058
	*p*-value	0.188	0.153	0.388	0.466	0.023	0.713	0.021	0.575
Cancer Therapy Satisfaction (CTSQ) ^8^									
Expectations of Therapy (ET)	rho	−0.123	−0.078	0.008	0.056	0.017	−0.174	−0.290 **	−0.120
	*p*-value	0.234	0.453	0.938	0.587	0.871	0.092	0.004	0.249
Feelings about Side Effects (FSE)	rho	−0.288 **	0.031	0.001	−0.040	0.100	0.141	−0.229 *	−0.101
	*p*-value	0.005	0.768	0.990	0.703	0.333	0.172	0.025	0.330
Satisfaction with Therapy (SWT)	rho	−0.373 **	0.099	−0.260 *	−0.148	0.276 **	−0.011	−0.171	−0.113
	*p*-value	0.000	0.339	0.011	0.152	0.007	0.914	0.097	0.275
Symptom Inventory (G-MDASI) ^9^	rho	0.251 *	−0.001	0.045	0.043	−0.291 **	0.022	0.127	−0.036
	*p*-value	0.014	0.995	0.666	0.682	0.004	0.835	0.220	0.726
Basic	rho	0.264 *	0.008	0.056	0.053	−0.326 **	−0.021	0.126	−0.081
	*p*-value	0.010	0.940	0.593	0.613	0.001	0.843	0.224	0.437
Interference items	rho	0.252 *	−0.013	0.069	0.032	−0.193	0.069	0.164	0.022
	*p*-value	0.014	0.902	0.506	0.761	0.061	0.503	0.113	0.832

^1^ (1: male, 2: female). ^2^ (years). ^3^ (1: married, 2: single, other). ^4^ (1: living with family members, 2: living alone). ^5^ (1: primary, 2: lower secondary, 3: upper secondary, 4: tertiary). ^6^ (1: Heraklion, 2: Other). ^7^ A higher score indicates higher fatigue. ^8^ A higher score indicates higher satisfaction. ^9^ A higher score indicates higher symptom intensity. * *p*-value < 0.05, ** *p*-value < 0.01.

**Table 6:** Adjusted odds ratios from multiple logistic regression analysis of high versus moderate/low CTSQ scores in relation to BFI-Gr, G-MDASI, and patient characteristics (n = 95).

	Cancer Therapy Satisfaction Questionnaire (CTSQ)					
	Expectations of Therapy (ET) ^1^		Feelings about Side Effects (FSE) ^2^		Satisfaction with Therapy (SWT) ^3^	
Prognostic Factors	OR	*p*-Value	OR	*p*-Value	OR	*p*-Value
Sex (female vs. male)	0.76	0.618	0.66	0.508	0.10	0.003
Marital status (other vs. married)	0.71	0.574	2.27	0.243	0.47	0.282
Level of education (1: primary, 2: lower secondary, 3: upper secondary, 4: tertiary)	1.04	0.869	0.93	0.821	1.62	0.166
Years from diagnosis (for each year of change)	0.83	0.161	0.96	0.853	0.91	0.557
G-MDASI (for each unit of change in the score of presence & intensity of symptoms)	0.99	0.970	0.45	0.018	0.85	0.499
BFI-Gr (for each unit of change in fatigue score)	0.96	0.050	0.98	0.470	0.97	0.374
pseudo R^2^ Negelkerke	0.242		0.360		0.449	
Multiple logistic regression analysis.						

^1^ Very high (75+) vs. medium/low (<75). ^2^ Very positive (75+) vs. negative (<75). ^3^ Very high (75+) vs. medium/low (<75).

## Data Availability

Data are available upon reasonable request due to privacy, ethical, and legal restrictions associated with human subject questionnaire data.
